# Shining a Light on Skeletal Muscle Regeneration: Red Photobiomodulation Boosts Myoblast Differentiation In Vitro

**DOI:** 10.1096/fj.202502477R

**Published:** 2025-10-29

**Authors:** Martina Parigi, Alessia Tani, Francesco Palmieri, Rachele Garella, Aurora Longhin, Gabriella Teti, Daniele Nosi, Daniele Guasti, Caterina Licini, Alessandra La Contana, Mirella Falconi, Monica Mattioli Belmonte, Roberta Squecco, Flaminia Chellini, Chiara Sassoli

**Affiliations:** ^1^ Department of Experimental and Clinical Medicine, Section of Anatomy and Histology, Imaging Platform University of Florence Florence Italy; ^2^ Department of Experimental and Clinical Medicine, Section of Physiological Sciences University of Florence Florence Italy; ^3^ Department of Biomedical and Neuromotor Sciences University of Bologna Bologna Italy; ^4^ Department of Clinical and Molecular Sciences (DISCLIMO) Università Politecnica Delle Marche Ancona Italy; ^5^ Department of Medical and Surgical Sciences University of Bologna Bologna Italy; ^6^ Advanced Technology Center for Aging Research, IRCCS INRCA Ancona Italy

**Keywords:** extracellular vesicles, HSP70, IL6, ion currents, laser, myogenesis, photobiomodulation, satellite cells, skeletal muscle

## Abstract

Although photobiomodulation (PBM) therapy (i.e., the application of light with a 600–1100 nm wavelength using laser or light‐emitting diode devices, a power density of less than 100 mW/cm^2^, and an energy density of less than 10 J/cm^2^ at the target) is emerging as a significant noninvasive strategy of promoting regeneration of damaged skeletal muscle tissue, its actual benefits remain debated. In particular, operating parameters exhibiting positive effects on regenerative muscle satellite stem cells need to be clearly identified. Hence, we investigated the effects of red PBM carried out by a laser diode (635 ± 10 nm; 0.4, 4, and 8 J/cm^2^; 4 mW/cm^2^; non‐contact mode; continuous wave; single exposure) on murine myoblasts undergoing differentiation and on mature myotubes by combining morphological, biochemical, and functional analyses. Red PBM, especially with a 4 J/cm^2^ energy density, did not alter cell viability but successfully promoted the expression of myogenic transcription factors as myoblast determination protein 1 (MyoD) and myogenin, as well as myotube formation, mitochondrial metabolism, and biogenesis. Consistently, electrophysiological analyses of cell membrane passive properties and inward ion currents indicated the acquisition of a more differentiated phenotype in PBM‐treated cells. Moreover, we found that PBM was able to enhance the release of extracellular vesicles (EVs) during cell differentiation according to a promyogenic phenotype. Red PBM treatment did not alter mature myotube viability and dimension while increasing their secretion of promyogenic EVs. Overall, this study provides experimental evidence supporting promyogenic effects of red PBM and the essential groundwork for further preclinical and clinical studies in the field of skeletal muscle regenerative medicine.

## Graphical abstract

Red (635 ± 10 nm) photobiomodulation (PBM) with an energy density of 4 J/cm^2^ enhances myogenic differentiation of murine skeletal myoblasts by promoting F‐actin assembly, modulating bioelectrical plasmamembrane properties, and increasing the expression of myogenic markers, mitochondrial biogenesis and activity, and secretion of promyogenic extracellular vesicles (EVs). Red PBM treatment does not alter mature myotubes' viability and dimension while increasing their secretion of promyogenic EVs.

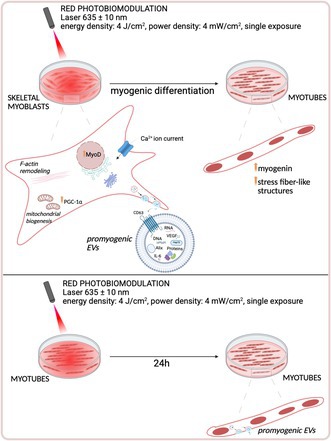

## 1 Introduction

Skeletal muscle accounts for nearly 40% of the human body mass and is responsible for a wide range of vital functions, including locomotion, force generation, energy storage, breathing, and metabolism [1]. Consequently, maintaining the homeostasis of skeletal muscles is fundamental to human health and well‐being. Several factors, such as trauma, infection, genetic defect, and aging, may affect muscle tissue structure and function, resulting in different outcomes based on the nature of the inciting factor. When acute/focal injuries occur, skeletal muscle is able to effectively regenerate the damaged tissue.

Muscle regeneration is largely attributed to a small population of resident stem cells, accounting for only ~2%–10% of the total myonuclei in healthy adult muscles (approximately from 2 × 10^5^ to 1 × 10^6^ cells/g muscle, depending on muscle and myofiber types), termed satellite cells (SCs), for their unique anatomical localization within close proximity to muscle fibers. Specifically, SCs are wedged between the basal lamina and sarcolemma of a mature myofiber, residing in a microenvironment known as a “niche” [2, 3, 4, 5]. In adult healthy muscle, SCs are in a quiescent state characterized by a lack of cell cycling and low metabolism, as well as low RNA levels. An injury or growth signals trigger their activation, enabling them to rapidly enter the cell cycle. Postnatal/adult SCs can be identified by the presence of the paired box trascription factor 7 (Pax7), which is expressed in quiescent and proliferating SCs and is recognized as their most reliable identifying marker [6]. In response to injury, the vast majority of SCs undergo asymmetric division to generate self‐renewing stem cells and myogenic progenitor cells that migrate into the damaged area, expand, and differentiate to repair the tissue [7]. The progression of SCs into the myogenic differentiation process is orchestrated by different myogenic regulatory factors (MRFs) including MRF4, myogenic factor 5 (Myf5), myoblast determination protein 1 (MyoD), and myogenin, functionally cooperating with Pax7, whose sequential expression and hierarchical relationship has been characterized. Generally, the earliest phase of myogenic commitment coincides with the upregulation of Myf5 activity, followed by the expression of MyoD, which marks the majority of activated and proliferating myoblasts. Subsequently, the downregulation of Pax7 and MyoD and the collateral upregulation of myogenin and MRF4 characterize the final adoption of the myogenic lineage by SCs into skeletal muscle progenitors/myocytes [8, 9, 10]. As a final step, myocytes fuse with each other to form polynucleated myotubes and then mature myofibers. They can also fuse with injured myofibers to restore them, providing new myonuclei and thus new genetic machinery. At this point, the expression of myosin heavy chain (MHC) and other contractile proteins starts to increase [8]. On the other hand, a small percentage of activated SCs undergo symmetric cell division, ensuing cells that withstand differentiation and are self‐renewed to maintain and replenish the pool of SCs over time [4, 8]. Noteworthy, the function and fate of SCs are finely regulated by cues coming from the niche including (among others) adhesion molecules, extracellular matrix (ECM) components, adenosine triphosphate (ATP) availability, growth factors, cytokines, physical factors such as oxygen tension, pH, and Ca^2+^ concentration of the environment and cell–cell interaction [8, 11].

In case of repetitive injuries, extensive muscle loss and/or chronic muscle damage mainly resulting from genetic defects, the tissue might not be completely morphofunctionally recovered. Indeed, SC functionality can be compromised and overwhelmed by the action of fibroblasts/myofibroblasts producing ECM components; as a result, injured muscle is often replaced by an aberrant fibro‐adipose tissue [12, 13].

Despite significant advancements in available technologies, conventional surgical, pharmacological, and physical rehabilitation treatments have some critical limitations, urging novel therapeutic approaches. Indeed, current treatments cannot not always guarantee rapid and complete tissue regeneration with effective morphofunctional recovery of a damaged muscle, particularly in cases involving persistent inflammation or severe and chronic muscle injury. In addition, there is no consensus on the most suitable treatment for muscle lesion management. A key challenge in the rehabilitation of muscle injuries is using therapeutic approaches that minimize fibrous scar tissue formation while simultaneously enabling the muscle to regenerate [14, 15]. In this context, photobiomodulation (PBM) has garnered attention due to its anti‐inflammatory, proregenerative, and antifibrotic effects in a variety of tissues, including skeletal muscle, improving tissue function [16, 17, 18, 19, 20, 21].

PBM, previously known as low‐level laser therapy (LLLT), is a non‐invasive non‐surgical phototherapy performing non‐thermal irradiation forms of light by laser and light‐emitting diodes (LED) operating in the red or infrared range of the light spectrum (wavelength 600–1100 nm) at a power density (irradiance) of less than 100 mW/cm^2^ and an energy density (fluence) generally of less than 10 J/cm^2^ at the level of the target tissue [22, 23]. Using the specific wavelengths and energy densities provided, light is able to easily penetrate tissues in a non‐destructive manner thereby modulating various cellular processes [23, 24, 25, 26, 27]. Currently, PBM is widely applied in different fields of medicine and is considered a safe technology [28, 29]. PBM offers a localized modality with minimal systemic risks. Despite this, results regarding PBM's effectiveness on skeletal muscle regeneration are controversial; moreover, operating parameters exhibiting positive effects on SCs need to be clearly identified [16, 18, 30, 31, 32, 33]. In such a context, the present in vitro study aimed to assess the effects of red PBM treatments on skeletal myoblasts undergoing differentiation and on differentiated myotubes by combining morphological, biochemical, and electrophysiological approaches. PBM was carried out in a single exposure with a red (635 ± 10 nm) diode laser by using three different energy densities of 0.4, 4, and 8 J/cm^2^. The rationale for testing these parameters essentially relies on the following considerations: (i) red light has been demonstrated to be effectively absorbed by cytochrome C oxidase, a key enzyme in the mitochondrial respiratory chain. This absorption triggers the activity of various molecules such as ATP, nitric oxide (NO), reactive oxygen species (ROS), and other signals that modulate skeletal muscle cell metabolic activity and differentiation [16, 34, 35, 36, 37]. Muscle cells possessing a high number of mitochondria are supposed to be particularly responsive to light [22]. (ii) Translationally, red light can effectively penetrate deep into the muscle tissue, considering that the oxygenated hemoglobin and myoglobin have relatively low absorption bands at wavelengths from 600 to 700 nm [23, 38, 39]. (iii) We tested three fluences, considering that a biphasic dose–response to the same wavelength is frequently observed, where lower light levels are often more effective than higher ones at stimulating cellular function [23]. (iv) Previous in vitro studies have demonstrated beneficial effects of red or infrared laser operating within the energy density range tested in this work, while exceeding approximately 15 J/cm^2^ can lead to a loss of these benefits or even to harmful effects [23, 40]. (v) Previous research from our group demonstrated the capability of red PBM to counteract the differentiation of fibroblasts into myofibroblasts [20, 41], the cell primarily responsible for fibrotic scar formation [42]. The demonstration of a promotion of myoblast differentiation by red PBM may contribute to providing essential experimental groundwork to give this therapy a double potential beneficial effect on a damaged skeletal muscle, both stimulating the intrinsic skeletal muscle regeneration process and concomitantly limiting the aberrant fibrotic reparative response necessary for effective functional recovery of a damaged muscle.

## 2 Materials and Methods

### 2.1 Cell Cultures

Murine myoblasts C2C12 cells, obtained by American Type Culture Collection (ATCC, Manassas, VA, USA, Cat# CRL‐1772, RRID:CVCL_0188), were cultured in a proliferation medium (PM) containing Dulbecco's modified Eagle's medium (DMEM; Sigma Aldrich, Milan, Italy) with 4.5 g/L glucose, supplemented with 10% fetal bovine serum (FBS; Sigma Aldrich), 1% penicillin/streptomycin (Sigma Aldrich), and incubated at 37°C in a humidified atmosphere of 5% CO_2_. When myoblasts reached a confluence of 70%–80%, they were induced to differentiate by replacing PM with a differentiation medium (DM) consisting of DMEM plus 2% horse serum (Invitrogen, CA, USA). PBM‐treated and ‐untreated cells were cultured for 24, 48, 72, and 96 h. In parallel, the conditioned media from cell cultures were collected for isolation and characterization of extracellular vesicles (EVs). For cell viability assay, cells were seeded in 96‐well culture plastic plates (Corning, Sigma Aldrich; Cat# CLS3367), for western blot (WB) and transmission electron microscopy (TEM) in 6‐well plates (Corning, Sigma Aldrich; Cat# 3516). For confocal laser scanning microscopy (CLSM) and electrophysiological analyses, cells were seeded on 20 × 20 mm glass coverslips (Exacta Optech Labcenter, Modena, Italy; Cat# ex‐9161019) placed into the wells of the 6‐well plates.

**FIGURE 1:**
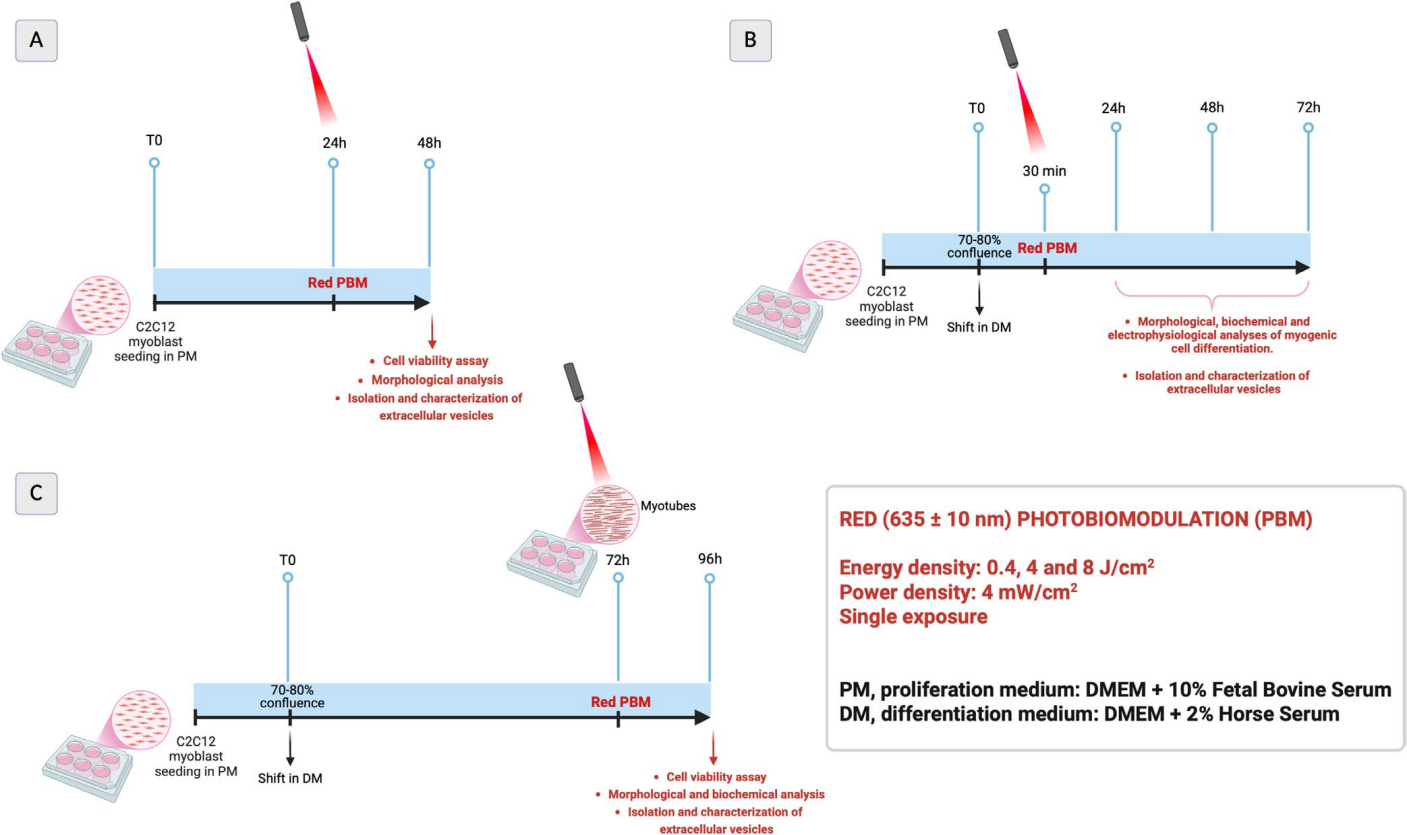
Experimental design (created with BioRender.com; RRID:SCR_018361).

The experimental design is depicted in Figure 1.

### 2.2 PBM Treatments

Laser characteristics and PBM parameters are summarized in Table 1. Specifically, C2C12 myoblasts plated in wells of 96‐well plates (well diameter: 0.6 cm, well surface area: 0.3 cm^2^) or 6‐well plates (well diameter: 3.5 cm, well surface area: 9.6 cm^2^) were irradiated at room temperature (RT) with a gallium–arsenide (GaAs) diode laser (GPUNI635, General Projects, Montespertoli, Florence, Italy) with a wavelength (λ) of 635 ± 10 nm (red) and exit in optical fiber, equipped with a focused fiber optic zoom handpiece, featuring a removable 60 mm spacer and an adjustable ring allowing beam diameters between 5 and 25 mm on the spacer plane. This zoom‐focused handpiece contains a system of collimation lenses that ensure a uniform “tophat” beam profile. The laser device was specially made for basic research purposes, and it is not commercially available (year of production: 2024; diode laser VIS 635 nm: class 4 according to the regulations CEI EN 60825‐1, max power output: 120 mW; max energy: 300 J). The irradiation was applied from the top, at a proper distance from the cells, resting the spacer on the plates and fixing the handpiece at a support stand, in order to cover the area of each well (for 96‐well plate, distance: 7.1 cm, laser beam diameter 0.6 cm; for 6‐well plates, distance: 7.8 cm, laser beam diameter 3.5 cm). Cells in PM were irradiated 24 h after seeding (Figure 1A) or 30 min after shifting in DM before being cultured in this medium for a further 24, 48, and 72 h (Figure 1B). In order to evaluate the effects of irradiation on mature myotubes, PBM was performed on C2C12 myoblasts induced to differentiate in DM for 72 h, a time point that enabled them to fuse with each other into myotubes. Analyses were conducted 24 h after treatment (96 h) (Figure 1C). Untreated cells were maintained under the same culture conditions as PBM‐treated ones.

**TABLE 1:** Laser specifications and PBM parameters.

Manufacturer	General Projects, Montespertoli, Florence, Italy					
Year produced	2024					
Type of emitters	Diode gallium–arsenide (GaAs) laser					
Wavelength and bandwidth (nm)	635 ± 10					
Pulse mode	Continuous wave					
Power density at target (mW/cm^2^)	4					
Application technique	Non‐contact mode					
Number and frequency of treatment sessions	Single exposure					
Energy density (J/cm^2^)	0.4		4		8	
Exposure duration (s)	100		1000		2000	
Area irradiated (cm^2^)	0.3	9.6	0.3	9.6	0.3	9.6
Power (mW)	1.2	38.4	1.2	38.4	1.2	38.4
Radiant energy (J)	0.12	3.84	1.2	38.4	2.4	76.8

To avoid overlapping or scattered irradiation, cells of each cell preparation were seeded in culture wells spaced apart: in the 96‐well plates, at least 4 empty wells separated each cell preparation; in the 6‐well plates, cells were plated in the first well (top left) and in the sixth well (bottom right). To minimize the influence of other light sources, PBM laser treatments were conducted in the dark. Experiments were carried out under a sterile hood. Eye protection of operators was assured by wearing safety glasses. Temperature was monitored during irradiation by using a thermal probe, and it remained unaltered significantly (≤ 0.1°C) during irradiation, thus excluding thermal effects.

### 2.3 Cell Viability Assay

Cell viability was determined by the 3‐(4.5‐dimethylthiazol‐2‐yl)‐5‐(3‐carboxymethoxyphenyl)‐2‐(4‐sulfophenyl)‐2H tetrazolium (MTS) assay (Promega Corp., Madison, WI). Cells were plated into 96‐well plates (initial density: 8 × 10^3^cells/well), with phenol red‐free culture media, subjected to PBM treatment or not and cultured as indicated in the experimental design (Figure 1A–C). After each culture time, the medium was replaced with 100 μL of a fresh one adding 20 μL of MTS test solution to each well. After 4 h of incubation, the optical density of soluble formazan deriving from the reduction of tetrazolium by mitochondrial enzymes of viable cells was measured at a wavelength of 492 nm using a multiwell scanning spectrophotometer (ELISA reader; Amersham, Pharmacia Biotech, Cambridge, UK).

### 2.4 Morphological Analyses

#### 2.4.1 Contrast Phase Light Microscopy, Myotube Morphology Analysis and Counting

Cellular morphological changes were monitored using a Nikon TMS inverted phase contrast microscopy with a 20× objective. High‐contrast images were acquired with a Canon EOS 450D digital camera. The quantification of the diameter of myotubes (cells containing a minimum of two nuclei) was performed in five photographs captured randomly for each experimental condition at 72 h (performed in triplicate). Measurements were carried out using ImageJ 1.49v software (https://imagej.net/ij/; RRID:SCR_003070). The diameter of each myotube was measured by drawing, at the nuclear region level, three different lines orthogonal to the longitudinal axis of the cell. The average of the three measures was considered as the mean diameter/width of each myotube.

The number of myotubes was counted in five randomly selected optical square fields (20× objective) under the Nikon TMS inverted phase contrast microscopy in each experimental condition at 48 and 72 h (performed in triplicate). Analyses were performed by two independent observers who were blinded to the culture conditions.

#### 2.4.2 Confocal Laser Scanning Microscopy (CLSM)

Cells on glass coverslips put on the bottom of 6 well‐plates were fixed with 0.5% buffered paraformaldehyde (PFA) for 10 min at RT. After permeabilization with cold acetone for 3 min, fixed cells were blocked with 0.5% bovine serum albumin (BSA; Sigma Aldrich) and 3% glycerol in phosphate‐buffered saline (PBS) for 20 min and then incubated overnight at 4°C in a humidified chamber, with the following primary antibodies: rabbit polyclonal anti‐MyoD (M‐318) (1:50; Santa Cruz Biotechnology, Santa Cruz, CA, USA, Cat# sc‐760, RRID:AB_2148870), mouse monoclonal anti‐myogenin (F5D) (1:50; Santa Cruz, Cat# sc‐12732, RRID:AB_627980), mouse monoclonal anti‐peroxisome proliferator‐activated receptor‐gamma coactivator (PGC)‐1α (1:100; Santa Cruz, Cat# sc‐518025, RRID:AB_2890187). Immunoreactions were revealed by incubation with specific anti‐mouse (1:200; Molecular Probes‐ Thermo Fisher Scientific, Eugene, OR, USA, Cat# A11001, RRID:AB_2534069) or anti‐rabbit Alexa Fluor 488‐conjugated (1:200; Molecular Probes, Cat# A11034, RRID:AB_2576217) for 1 h at RT. In other experiments, cells were stained with Phalloidin‐TRITC (1:100; Sigma Aldrich, Cat# P1951, RRID:AB_2315148) to reveal actin filament organization. Nuclei were counterstained with propidium iodide (PI, 1:200; Molecular Probes, Cat# P1304MP, at room temperature for 2 min). MitoTracker Red CMXRos (100 nM, Molecular Probes, Cat# M7512, for 30 min) was used to stain mitochondria in living cells as reported previously [43].

Observations were conducted using a confocal Leica TCS SP5 microscope equipped with a HeNe/Ar laser source for fluorescence measurements and differential interference contrast (DIC) optics, with a Leica Plan Apo 63X/1.43 NA oil immersion objective (Leica Microsystems, Mannheim, Germany). A series of optical sections (1024 × 1024 pixels each; pixel size 204.3 nm) were captured throughout the depth of the specimens at intervals of 0.4 μm (z‐step size) and then projected onto a single “extended focus” image. Quantitative analysis of MyoD and myogenin expressing nuclei was performed by counting positive nuclei in a minimum of 5 random 200 × 200 μm^2^ microscopic fields (63× objective) for each experimental group, and the results were reported as the percentage of positive nuclei of the total cell nuclei. Densitometric analyses of fluorescent signal intensity for each specific marker (TRITC‐labeled phalloidin/F‐actin, MitoTacker/mitochondria, Alexa Fluor 488/PGC‐1α) was performed by using ImageJ 1.49v software (https://imagej.net/ij/) on digitized images: 10 regions of interest (ROI; 100 μm^2^) were selected in 8 cells for each confocal stack (10 for each experimental point performed in duplicate). Experiments were conducted in triplicate (*n* for each experimental point = ROI = 100; number of examined cells for each experimental point = 80).

The fusion index, commonly used in myoblast culture assays to assess the amount of their fusion to form syncytial myotubes and thus a key indicator for quantifying the differentiation of a myoblast population [44], was calculated as the ratio of nuclei number in myotubes (containing ≥ 2 nuclei) and the total number of nuclei present in a microscopic field of view (200 × 200 μm^2^) and expressed as a percentage.

Five random fields were analyzed for each experimental condition by two different investigators (each experimental condition was performed in triplicate).

#### 2.4.3 Transmission Electron Microscopy (TEM)

For TEM analyses, murine C2C12 myoblasts (pellet) were fixed in Karnovsky's fixative overnight at 4°C, post‐fixed in 1% OsO_4_ in 0.1 M phosphate buffer (pH 7.4) for 1 h at RT, dehydrated in a graded acetone series, passed through propylene oxide, and then embedded in Epon 812 (Sigma‐Aldrich, Cat# 45345). Ultrathin sections (60 nm thick) were contrasted with UranyLess EM stain (Electron Microscopy Sciences, Foster City, CA, USA, Cat# 22409) and alkaline bismuth subnitrate and then examined using a Jeol 1010 electron microscope (Jeol, Tokyo, Japan) at 80 kV equipped with a Jeol Veleda high‐resolution digital camera (Veleta, EMSIS GmbH, Münster, Germany).

#### 2.4.4 Machine Learning Morphometric Analysis of Mitochondria

Quantification of total mitochondrial cristae length and mitochondrial surface area, and of the ratio between these two values relatable to metabolic activity, was carried out in two sequential steps using TEM images captured at 50 000× magnification (image resolution: 2048 × 2048 pixels; pixel scale: 1.38 pixels per nanometer). Image processing and analysis were performed utilizing AIVIA machine learning software (https://www.leica‐microsystems.com/it/prodotti/software‐per‐microscopi/p/aivia/).

The initial phase relied on the Cellpose Enhancement tool, which applies pre‐trained deep learning models to overcome two primary challenges. In the first place, the acquired images exhibited variability in quality, and then, mitochondria displayed differences in matrix density. These factors complicated the direct application of machine learning models for mitochondrial membrane identification, as these structures were often indistinguishable from membranes of different organelles, such as the rough and smooth endoplasmic reticulum. The Cellpose Enhancement tool allowed the identification of objects within the images based on parameters such as size and sphericity. To this purpose, an average object diameter between 110 and 150 pixels (equivalent to 151.72 to 206.90 nm) was defined. Further refinement of the object selection process was achieved by adjusting the percentile of the probability map, which was set between the 92nd and 97th percentiles depending on both image quality and mitochondrial matrix density. The identified mitochondrial sections were enclosed within defined ROIs, and their respective areas were measured. In the subsequent phase, mitochondrial cristae were segmented within the previously delineated ROIs using specifically trained machine learning models. This segmentation allowed the quantification of cristae length. The relationship between the total cristae length and the mitochondrial area was expressed through the C coefficient, defined as:

C=Total cristae length/Mitochondrial area

### 2.5 Western Blot (WB)

Total proteins from cell lysates were extracted and quantified as previously reported [45]. Forty micrograms of total proteins were electrophoresed on NuPAGE 4%–12% Bis‐Tris Gel (Thermo Fisher Scientific, Waltham, MA, USA; 200 V, 40 min) and blotted onto polyvinylidene difluoride (PVDF) membranes (Thermo Fisher Scientific; 30 V, 1 h). First, membranes were incubated on a rotary shaker for 30 min at RT with the Blocking Solution provided in the Western Breeze Chromogenic Western Blot Immunodetection Kit (Thermo Fisher Scientific) and then overnight at 4°C with the following antibodies: rabbit polyclonal anti‐MyoD (M‐318) (1:500; Santa Cruz, Cat# sc‐760, RRID:AB_2148870), mouse monoclonal antimyogenin (F5D) (1:500; Santa Cruz), monoclonal mouse anti‐PGC‐1α (1:1000; Santa Cruz); mouse monoclonal anti‐α‐tubulin (DM1A) (1:2500; Santa Cruz, Cat# sc‐32293, RRID:AB_628412). Protein immunodetection was performed according to the Western Breeze Chromogenic Immunodetection protocol (Thermo Fisher Scientific). Densitometric analysis of the bands was performed using ImageJ 1.49v software (https://imagej.net/ij/), and values were normalized to α‐tubulin for each result, assuming α‐tubulin as a control invariant protein.

### 2.6 Electrophysiological Recordings

Electrophysiological analyses were achieved by the whole‐cell patch clamp technique. Cells plated on a glass coverslip, previously subjected to different experimental conditions, were placed in a bath chamber arranged on the stage of a Nikon Eclipse TE200 inverted microscope (Nikon Europe BV, 1076 ER Amsterdam, The Netherlands) constantly superfused at a rate of 1.8 mL min^−1^ with physiological external solution (mM): 150 NaCl, 5 KCl, 2.5 CaCl_2_, 1 MgCl_2_, 10 D‐glucose, and 10 HEPES (pH 7.4 with NaOH). Starting from borosilicate glass capillaries (GC150‐7.5; Clark, Electromedical Instruments, Reading, UK) we made the patch pipettes by means of a vertical puller (Narishige, Tokyo, Japan). Patch pipettes were then filled with a standard internal solution having the following composition (mM): 130 KCl, 10 NaH_2_PO_4_, 0.2 CaCl_2_, 1 EGTA, 5 MgATP, and 10 HEPES (pH 7.2 with KOH). Once filled, the pipette resistance was 3–7 MΩ. The apparatus for the electrophysiological measurements consisted of the Axopatch 200 B amplifier, A/D‐D/A interfaces Digidata 1200; Pclamp 6 software (RRID:SCR_018866, Axon Instruments, Foster City, CA, USA) as described previously [46, 47]. In the current clamp mode of the 200 B amplifier, we recorded the resting membrane potential (RMP) by using a stimulus I = 0. In voltage‐clamp mode, we assessed the passive membrane properties by the application of a voltage pulse of ±10 mV starting from a holding potential (HP) = −70 mV. We analyzed our records by the Clampfit 9 software (Axon Instruments), and the decay of the recorded passive current was fitted by the sum of 2 exponential functions representing the time course of the surface and tubular membrane passive current [48]. The linear capacitance, Cm, was estimated from the area beneath the capacitive transient current. Since the membrane‐specific capacitance is assumed to be 1 μF/cm^2^, the Cm value can be considered as an index of the cell surface area. The specific membrane conductance, indicating the membrane permeability, was determined by the ratio Gm/Cm, where Gm (membrane conductance) corresponded to 1/Rm, where Rm is the membrane resistance. Rm was calculated from the steady‐state membrane current (Im) as described previously [49]. To record transmembrane ion currents, we used the voltage‐clamp mode. In particular, to record only Ca^2+^ current, I_Ca_, we used a Na^+^‐ and K^+^‐free high‐TEA external solution (mM): 10 CaCl_2_, 145 tetraethylammonium bromide, 10 HEPES, and a suitable filling pipette solution (mM): 150 CsBr, 5 MgCl_2_, 10 ethylene‐bis (oxyethylenenitrilo) tetraacetic acid (EGTA), and 10 (4‐(2‐hydroxyethyl)‐1‐piperazineethanesulfonic acid) (HEPES) (pH = 7.2) [50]. A pulse protocol of stimulation consisting of 1‐s step voltage pulses, ranging from −80 to 50 mV, in 10 mV increments, was applied from HP = −80 mV to elicit I_Ca_. In any case, the capacitive and leak currents were removed online by the P4 procedure. To properly compare the currents evoked from myotubes of different dimensions, we normalized the current amplitude values to Cm. The ratio I/Cm (pA/pF) represents the current density. All drugs were from Merck Life Sciences (Burlington, MA, USA). The electrophysiological experiments were achieved at room temperature (about 22°C).

### 2.7 Isolation and Characterization of Extracellular Vesicles (EVs)

#### 2.7.1 Isolation of EVs

A 40% (w/v) stock solution of polyethylene glycol (PEG 8000; Sigma Aldrich) was prepared in Dulbecco's PBS, filtered through a 0.2 μm membrane (Sarstedt, Numbrecht, Germany), and stored at 4°C protected from light. Cell culture media collected after each treatment (Figure 1A–C) were centrifuged at 900 g for 30 min. At the end, the supernatants were filtered with 0.4 mm filters (Sarstedt) and mixed with a 12% (w/v) PEG 8000 solution in a 3:1 ratio by vortexing and incubating overnight at 4°C. Then, the EVs were spun down by centrifugation at 10 000 g and 4°C for 1 h. The obtained pellets were resuspended in PBS solution and stored at 4°C or directly stored at −80°C until protein extraction.

#### 2.7.2 Morphological Evaluation by TEM

For TEM analysis, 10 μL of EVs solution in PBS was mounted on Formvar/carbon film‐coated mesh nickel grids (Electron Microscopy Sciences) for 20 min avoiding air dehydration. Then, the excess liquid was removed with filter paper, and 10 μL of 1% uranyl acetate was added onto the grids. Stained grids were then observed by transmission electron microscope CM10 Philips (FEI Company, Eindhoven, The Netherlands) at an accelerating voltage of 80 kV. Images were recorded by Megaview III digital camera (FEI Company, Eindhoven, The Netherlands).

#### 2.7.3 Biochemical Characterization by WB

EVs characterization was performed by WB detecting the expression of markers, namely, tetraspanin CD63, ALG‐2 interacting protein X (Alix), and Heat Shock Protein (HSP70) in accordance with MISEV (Minimal Information for Studies of Extracellular Vesicles) guidelines [51, 52, 53, 54] and the promyogenic factors, namely, interleukin‐6 (IL‐6) [55, 56] and vascular endothelial growth factor (VEGF) [57, 58]. Total protein was extracted using a radioimmunoprecipitation assay (RIPA)‐modified lysis buffer (Pierce, Thermo Fisher Scientific, Monza, Italy) supplemented with 25 μmol/L protease inhibitor cocktail (Pierce, Thermo Fisher Scientific) and 1 μL of β‐mercapto‐ethanol (Sigma Aldrich). The amount of protein obtained from each sample was quantified by Bradford assay (Sigma Aldrich), and 10 μg of total protein was separated by 4%–12% sodium dodecyl sulfate polyacrylamide gel electrophoresis (SDS‐PAGE), followed by transfer onto a nitrocellulose membrane (GE Healthcare, Amersham, UK). Subsequently, the membranes were incubated with 5% BSA (Sigma Aldrich) or no fat milk (blocking reagent) to remove the non‐specific binding proteins, followed by incubation with the primary antibodies: mouse anti‐tetraspanin CD63 (TS63) (1:1000; Invitrogen, Thermo Fisher Scientific, Cat# 10628D, RRID:AB_2532983), rabbit anti‐Alix (E6P9B) (1:1000; Cell Signaling Technology, Cat# 92880, RRID:AB_2800192), rabbit anti‐HSP70 (1:1000; Cell Signaling Technology, Cat# 4872, RRID:AB_2279841), rabbit anti‐VEGF (1:1000; Thermo Fisher Scientific, Cat# PA5‐16754, RRID:AB_10979267), rabbit anti‐IL‐6 (1:1000; Invitrogen, Thermo Fisher Scientific, Cat# 701028, RRID:AB_2532352). All the primary antibodies were in blocking reagent at 4°C, overnight. After being washed with TRIS‐buffered saline (TBS)‐tween buffer, samples were incubated with horseradish peroxidase (HRP)‐linked anti‐rabbit IgG secondary antibody (1:10 000; Cell Signaling Technology, Cat# 7074, RRID:AB_2099233) or anti‐mouse IgG secondary antibody (1:20 000; Sigma Aldrich, Cat# AP308P, RRID:AB_92635), both diluted in TBS‐tween buffer at RT for 90 min. The antibody signal was visualized by an enhanced chemiluminescence system (Pierce, Thermo Fisher Scientific). Images were obtained by using IBright Western Blot Imaging System. The densitometric analysis was performed using ImageJ software (https://imagej.net/ij/). The intensities of the specific protein bands were corrected for total protein staining, and they were expressed as relative value compared to the intensity of the respective control sample. The experiments were performed in triplicate.

### 2.8 Statistical Analyses

The statistical analysis was made with Microsoft Excel (Microsoft, Washington, USA, RRID:SCR_016137). Values are expressed as mean ± SD. The Shapiro–Wilk test online was used to assess the normal distribution of data. Student's *t*‐test was used to compare two experimental groups; One‐way or Two‐way Analysis of Variance (ANOVA) was used for multiple comparisons with Bonferroni post hoc correction or post hoc Tukey HSD test calculator (https://astatsa.com/OneWay_Anova_with_TukeyHSD/). Statistical significance was set at *p* < 0.05. Graphs were generated with Microsoft Excel.

## 3 Results

### 3.1 Effect of Red PBM Treatments on Cell Viability, Myotube Formation, F‐Actin Cytoskeleton and Myogenic Markers' Expression

**FIGURE 2:**
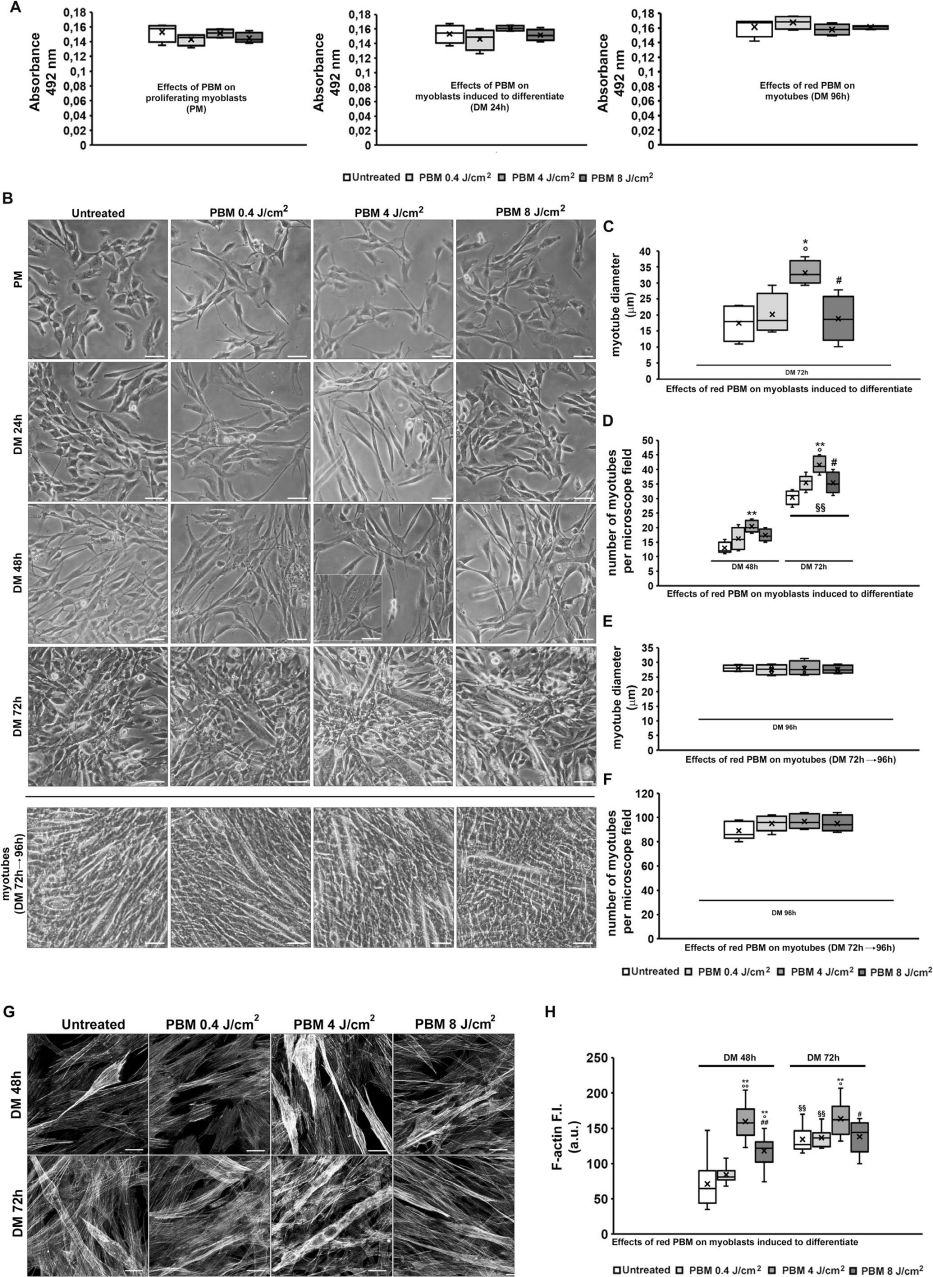
Cell viability, morphology, and cytoskeleton assembly. (A) MTS assay performed in myoblasts cultured in proliferation medium (PM, left graph), in myoblasts subjected to red PBM treatments with different energy density and induced to differentiate for 24 h in differentiation medium (DM, middle graph) and on differentiated myotubes (72 h) subjected to PBM and cultured for additional 24 h (96 h, right graph). Cells not subjected to PBM were indicated as untreated. (B) Contrast phase light microscopy of myoblasts induced to differentiate in DM for 24, 48 or 72 h subjected or not (untreated) to the three red PBM with different energy density and (last row) of myotubes subjected to PBM after 72 h in DM and cultured for additional 24 h (96 h). Scale bar: 50 μm. (C, D) Quantitative analyses of the mean myotube diameter and the number of myotubes per microscopic field in cells undergoing differentiation. (E, F) Quantitative analyses the mean myotube diameter of myotubes (96 h). (G) Representative confocal laser scanning microscopy (CLSM) fluorescence images of cells undergoing differentiation for the indicated times subjected or not (untreated) to red PBM treatments stained with phalloidin‐TRITC (gray, pseudocolor) to show F‐Actin organization. Scale bar: 15 μm. (H) Densitometric analyses of the mean fluorescent signal intensity (F.I.) of TRITC‐labeled phalloidin/F‐Actin expressed in arbitrary units (a.u.). Data are mean ± SD. Statistical Significance in C, D, H: *p < 0.05, **p < 0.01 vs. untreated; °*p* < 0.05 vs. PBM 0.4 J/cm^2^; ^#^ *p* < 0.05 vs. PBM 4 J/cm^2^, ^§§^ *p* < 0.01 vs. 48 h (One‐way ANOVA with post hoc Tukey HSD).

In order to determine the potential cytotoxic effect of the three red PBM treatments (0.4, 4, and 8 J/cm^2^), we first evaluated cell viability by MTS assay. C2C12 myoblasts were cultured in proliferating conditions (PM) for 24 h or in differentiating ones (DM) for 24 h when they reached confluence. Cells were subjected to PBM treatments 24 h after cell seeding in PM (Figure 1A) or 30 min after shifting in DM (Figure 1B). The analysis showed that the three red PBM treatments did not perturb the viability of C2C12 myoblasts cultured in PM or in DM for 24 h (Figure 2A). None of the tested PBM treatments induced significant morphological alterations in cells cultured in PM, as monitored by phase‐contrast microscopy (Figure 2B first row).

The myogenic differentiating capability of myoblasts cultured in DM was initially monitored by phase‐contrast microscopy (Figure 2B). As expected, untreated cells (serving as internal control) underwent typical morphological changes as a result of shifting from PM to DM and during differentiation (Figure 2B, first column): cells cultured in DM for 24 h were more elongated than those cultured in PM, which appeared star‐shaped. After 48 h of culture in DM, they started aligning and fusing with each other to form multinucleated syncytial tubular myotubes that became clearly visible after 72 h. Cells underwent red PBM treatment with energy densities of 0.4 and 8 J/cm^2^ responded in the same way as the untreated ones (Figure 2B second and fourth columns, C, D). Of note, red PBM with 4 J/cm^2^ performed on cells in DM (Figure 1B) turned out to be able to stimulate muscle cell differentiation (Figure 2B third column). Indeed, compared to control, PBM‐treated cells in DM appeared more elongated after 24 h, and we were able to appreciate myotube formation after 48 h of differentiation (see inset in Figure 2B, third column), featuring myotubes boosted in number and size (Figure 2C,D).

**FIGURE 3:**
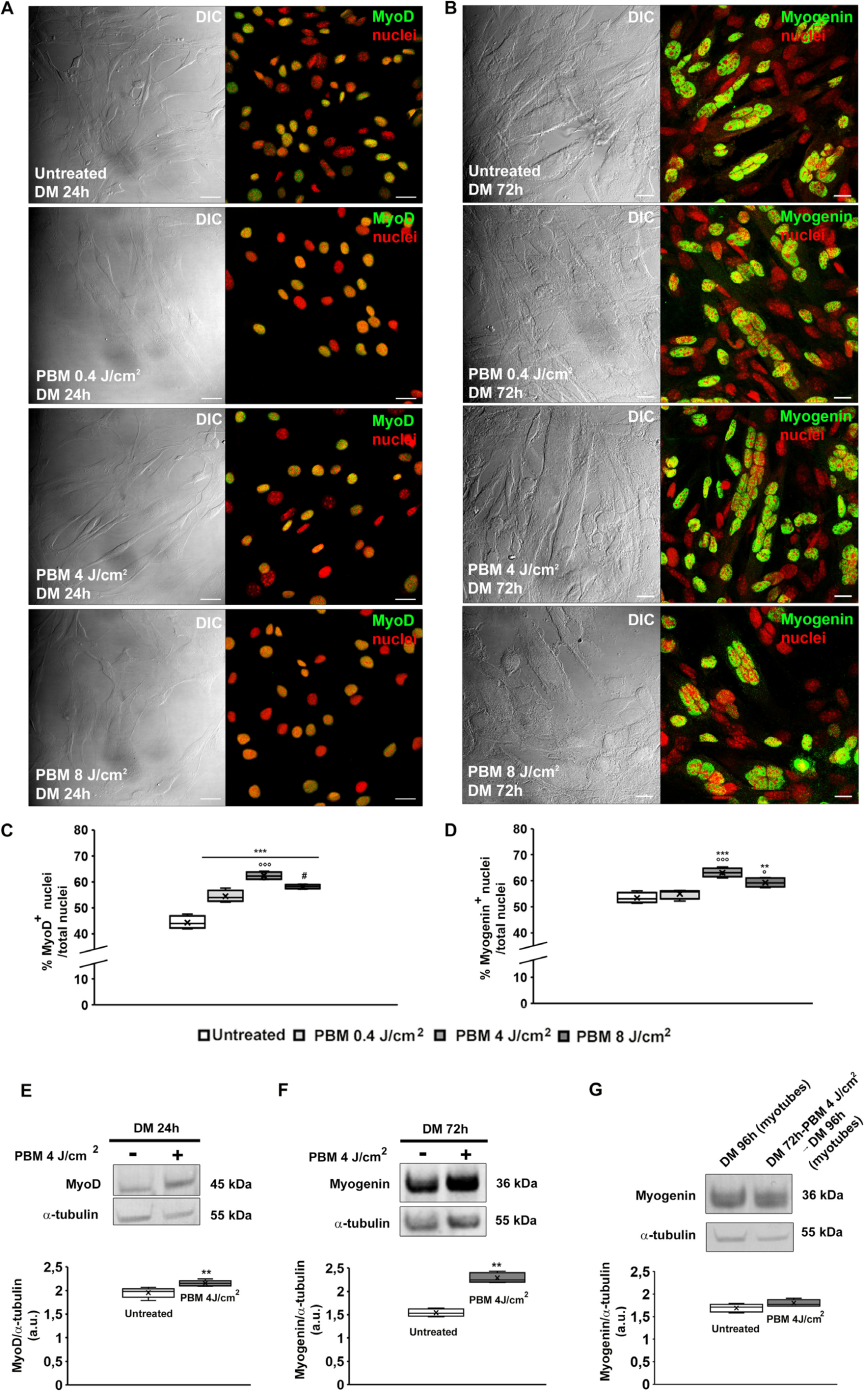
Expression of myogenic markers. (A, B) Representative differential interference contrast (DIC, gray) and confocal laser scanning microscopy (CLSM) fluorescence images of myoblasts subjected or not (untreated) to red PBM treatments with different energy density (0.4, 4, 8 J/cm^2^) and induced to differentiate in differentiation medium (DM) for the indicated times, immunostained with antibodies against the myogenic differentiation markers (A) MyoD and (B) myogenin (in green); nuclei are counterstained with propidium iodide (red). Scale bar 25 m. (C, D) Quantitative analyses of MyoD and myogenin positive nuclei expressed as the percentage of the total nuclei, respectively. (E–G) Western blotting analysis of (E) MyoD and (F) myogenin of myoblasts subjected or not (untreated) to red PBM treatment with 4 J/cm^2^ energy density and undergoing differentiation for the indicated times and (G) of myogenin of myotubes (cells were cultured in DM for 72 h in order to obtain myotubes and at this time point the myotubes were subjected or not to red PBM, cultured in DM for additional 24 h and analyzed (DM 96 h). Representative blot and bar charts showing the densitometric analysis of the bands normalized to α‐tubulin. a.u.: arbitrary units. Data are mean ± SD. Statistical significance in C, D, E: **p < 0.01, ***p < 0.001 vs. untreated; °*p* < 0.05, °°°*p* < 0.001 vs. PBM 0.4 J/cm^2^; ^#^ *p* < 0.05 vs. PBM 4 J/cm^2^ (Student's *t*‐test with post hoc Tukey HSD).

The myogenic differentiation was also evaluated by analyzing cytoskeleton organization, specifically F‐actin networks/structures (Figure 2G,H) and the expression of the typical early and late myogenic markers such as MyoD and myogenin (Figure 3). As shown by CLSM analysis, the untreated differentiating cells/myotubes exhibited the organization of F‐actin filaments into well‐assembled stress fiber‐like structures parallelly aligned along the cytoplasm, increasing during differentiation (Figure 2G,H). Of note, consistent with a thickening of these filamentous structures, the fluorescent signals were more intense in cells subjected to PBM with an energy density of 4 J/cm^2^.

CLSM immunofluorescence analyses revealed cells with MyoD‐positive nuclei after 24 h of culture in DM (Figure 3A,C) and myotubes with myogenin‐positive nuclei after 72 h (Figure 3B,D). A significant increase in the percentage of nuclei positive for MyoD and myogenin was especially observed after red PBM treatment with an energy density of 4 J/cm^2^. Results of the fusion index quantification confirmed the capability of red PBM to promote myotube formation: DM 72 h, 22.46% ± 12.7%; DM + PBM 0.4 J/cm^2^ 21.24% ± 11.4%; DM + PBM 4 J/cm^2^ 40.43% ± 19.8%; DM + PBM 8 J/cm^2^ 15.31% ± 8.1%; (*p* < 0.05 4 J/cm^2^ vs. untreated, *p* < 0.05 4 J/cm^2^ vs. 0.4 J/cm^2^, *p* < 0.01 8 J/cm^2^ vs. 4 J/cm^2^). WB analyses of MyoD (Figure 3E) and myogenin (Figure 3F) expression were consistent with CLSM results. Finally, red PBM did not alter the viability and morphology of differentiated myotubes (Figures 1C, 2A right graph, 2B last row, 2E,F), as well as myogenin expression (Figure 3G). In this set of experiments, cells were cultured in DM for 72 h in order to obtain myotubes, and at this time point, the myotubes were subjected or not (untreated) to red PBM, cultured in DM for an additional 24 h, and analyzed (DM 96 h).

Overall, these results indicate that none of the red PBM treatments induced cytotoxic effects, and especially the PBM treatment performed with an energy density of 4 J/cm^2^ promoted differentiation of myogenic cells.

### 3.2 Functional Effects of Red PBM on Plasmamembrane Bioelectrical Properties

**FIGURE 4:**
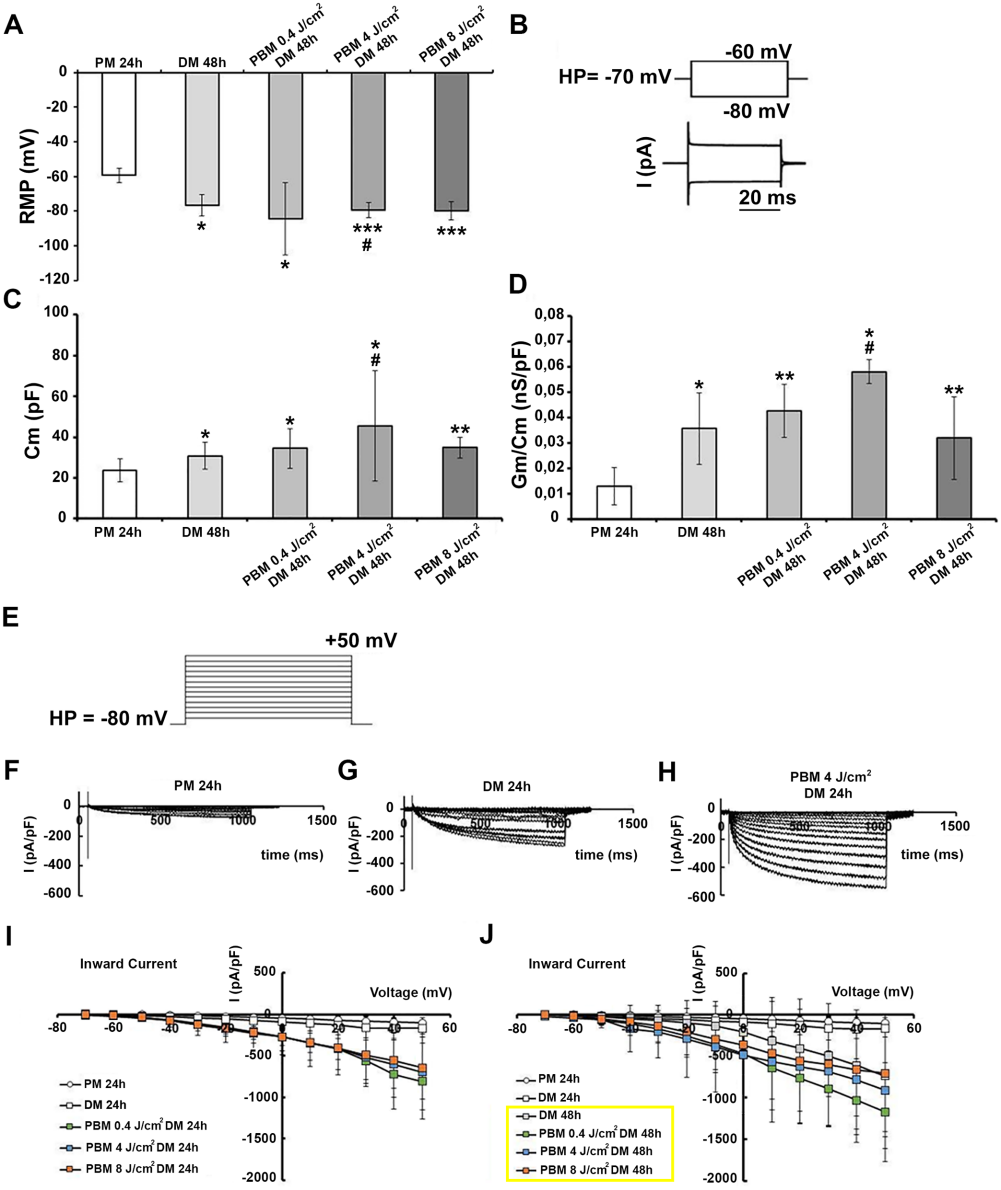
Effect of PBM on cell membrane features: passive properties and inward currents. Myoblasts exposed or not (untreated) to red PBM with three different energy densities (0.4, 4, 8J/cm^2^) were cultured in proliferation medium (PM) or in differentiation medium (DM) for 24 or 48 h. (A) Resting membrane potential (RMP, in mV) evaluated in current clamp mode (I = 0 nA). (B) Pulse protocol of stimulation (top) in voltage clamp to evoke passive currents (bottom, I in pA). (C) Cell capacitance (Cm, in pF). (D) Specific membrane conductance (Gm/Cm in nS/pF) as an index of membrane permeability. Data are mean ± SD. *p < 0.05, **p < 0.01; ***p < 0.001 vs. PM; ^#^ *p* < 0.05 vs. DM 48 h (One‐way ANOVA with Bonferroni correction) (*n* = 5–11). (E) Pulse protocol of stimulation in voltage clamp to evoke ion currents. (F–H) Representative current records (I, in pA/pF) evoked in C2C12 cells in the indicated conditions. (I, J) Overall I‐V plots of the current amplitude normalized to Cm (I, in pA/pF) as a function of voltage pulses related to all the experiments done at 24 h (I) and 48 h (J). Data are mean ± SD. (*p* > 0.05, Two‐way ANOVA with Bonferroni correction) (*n* = 3–7).

In parallel, we achieved a functional characterization of the murine myoblasts by whole‐cell patch‐clamp electrophysiological technique (Figure 4). Again, we observed that the treatment of red PBM with a 4 J/cm^2^ energy density helped the cells acquire a more differentiated myogenic phenotype better than the DM alone. For the bioelectrical properties, this was particularly evident after 48 h in DM, and data related to the differentiation at this time point are reported in Figure 4A–D. In particular, the analysis of resting membrane potential (RMP) (Figure 4A) showed, as expected, a statistically significant hyperpolarization in cells cultured in DM compared to PM. This behavior was even significantly enhanced by red PBM, especially with 4 and 8 J/cm^2^ energy density. However, only the energy density of 4 J/cm^2^ was effective in inducing a statistically significant hyperpolarization compared to cells cultured in DM and not subjected to PBM.

The analysis of the passive properties was made applying a suitable step pulse protocol (Figure 4B). The resulting cell capacitance, Cm, showed a significant increase shifting from PM to DM (Figure 4C), suggesting a surface membrane enlargement. This increase was also observed for PBM‐treated cells, but as previously noted, only cells irradiated with red PBM 4 J/cm^2^ showed a statistically significant increase of Cm compared to nonirradiated cells. A similar differentiating trend was observed for the specific membrane conductance Gm/Cm (Figure 4D): cells cultured in DM showed increased values compared to those in PM; in particular, those irradiated with red PBM 4 J/cm^2^ showed a statistically significant increased value compared to not‐irradiated cells in DM.

Then, aiming to estimate another electrophysiological aspect of myoblastic differentiation, we tested the eventual occurrence of Ca^2+^ ion current (I_Ca_), which normally plays a pivotal role in excitation–contraction (EC) coupling in mature skeletal muscle fibers [59]. By applying the voltage pulse protocol displayed in Figure 4E, C2C12 myoblasts cultured in PM elicited a very small‐amplitude inward current (Figure 4F), typical of undifferentiated cells. This current tended to increase during differentiation, as shown for a representative cell cultured in DM for 24 h (Figure 4G), especially when the cells in this condition were exposed to red PBM treatment (Figure 4H). The overall effect of red PBM treatment at different energies estimated for two different times (24 and 48 h) in DM is revealed in Figure 4I,J, respectively. In particular, the I‐V plot showed that the increase of the inward current amplitude was almost linear with the voltage step applied, indicating scarce voltage dependence likely due to the short differentiation time. However, as already appreciable after 24 h in DM, all the red PBM treatments tended to enhance the current amplitude (Figure 4I), even if the differences between the effects due to the three different energy densities are negligible. After 48 h in DM (Figure 4J), the current amplitude further increased as expected (compare data related to DM 24 h vs. DM 48 h). Again, this trend was enhanced by red PBM treatment, although with no significant differences between the energy densities. Notably, when cells were cultured in DM, the normalized current amplitudes obtained after red PBM did not have a strictly linear distribution with voltage, indicating the possible appearance of more voltage‐dependent calcium channels in this experimental condition.

Based on the above morphofunctional data demonstrating the best efficacy of red PBM with 4 J/cm^2^ energy density in enhancing myogenic differentiation, successive experiments were aimed to deeper investigate the effects of such treatment.

### 3.3 Red PBM (4 J/cm^2^) Promotes Mitochondrial Biogenesis and Metabolic Activity

**FIGURE 5:**
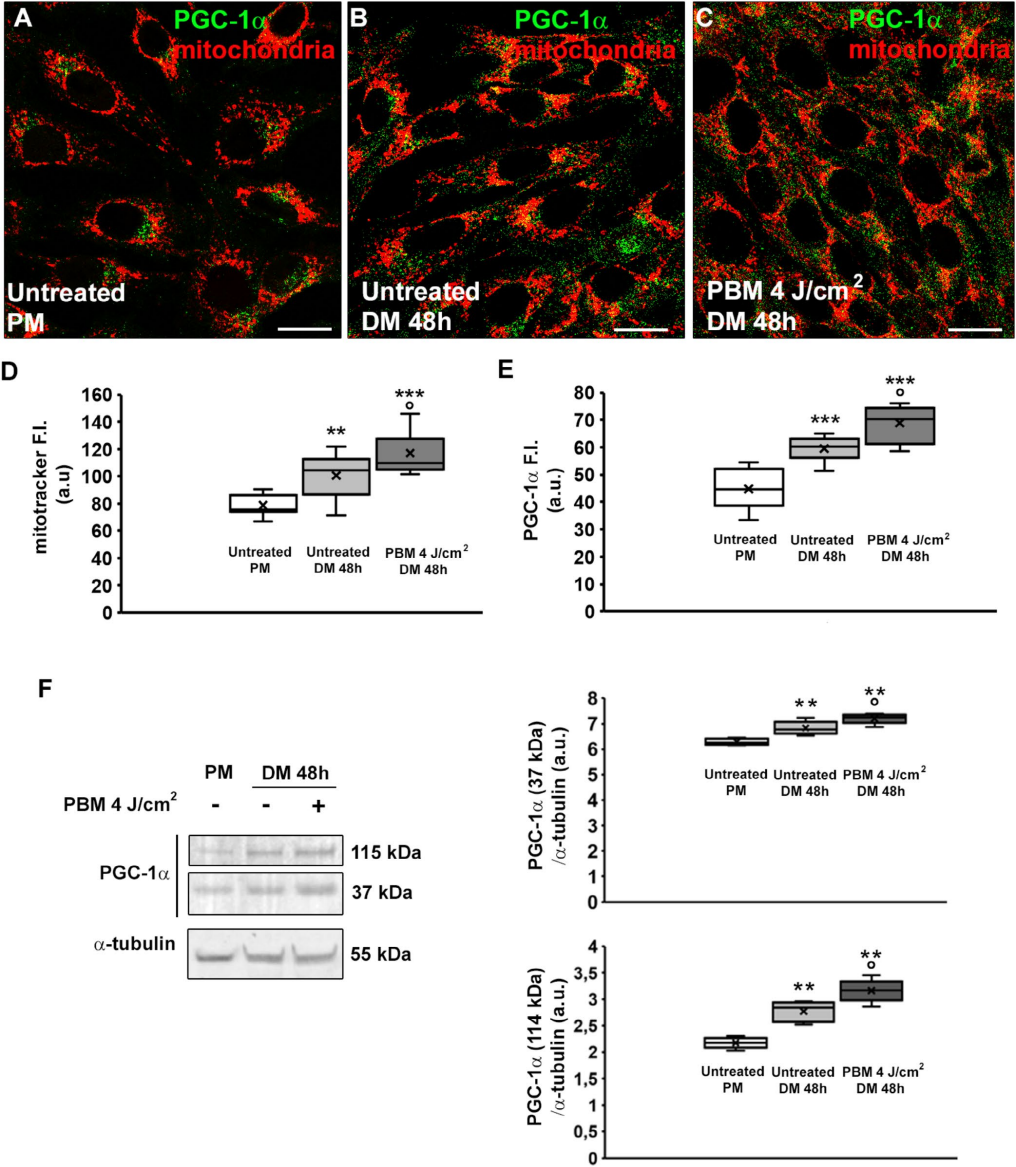
Analyses of mitochondria and of peroxisome proliferator‐activated receptor‐gamma coactivator (PGC)‐1α expression. Myoblasts were cultured in proliferation medium (PM) or exposed or not (untreated) to PBM with 4 J/cm^2^ energy density and then cultured in differentiation medium (DM) for 48 h. (A–C) Representative confocal laser scanning microscopy (CLSM)fluorescence images of myoblasts labeled with MitoTracker to mark mitochondria (red) and immunostained for PGC‐1α expression (green). Scale bar: 20 μm. (D, E) Densitometric analyses of the fluorescence intensity (F.I.) of mitochondria and PGC‐1α respectively, expressed in a.u. (arbitrary units). (F) WB analysis of PGC‐1α in the indicated conditions: representative blot and bar charts showing the densitometric analysis of the bands normalized to α‐tubulin. Molecular weight of full length of PGC‐1α, 115 kDa, molecular weight of N‐terminal truncated NT‐PGC‐1α: 37 kDa. a.u.: arbitrary units. Data are mean ± SD. **p < 0.01, ***p < 0.001 vs. untreated PM; °*p* < 0.05 vs. untreated DM 48 h (One‐way ANOVA with post hoc Tukey).

We next analyzed the impact of red PBM with 4 J/cm^2^ energy density on mitochondria with the aim of gathering further mechanistic insight. As judged by CLSM analysis, myoblasts cultured in DM for 48 h exhibited an increase in the intensity of the MitoTracker dye signal, used to label mitochondria, as well as of PGC‐1α (Figure 5A,B,D,E) regarded as a marker of mitochondrial biogenesis [60].

Of note, the intensity of the fluorescent signals of both mitochondria and PGC‐1α increased in cells induced to differentiate after red PBM treatment (Figure 5C–E). WB analysis of PGC‐1α confirmed the morphological data (Figure 5F) showing after PBM irradiation an increase of both the full‐length protein (114 KDa) and N‐terminal truncated protein (37 KDa) [61].

**FIGURE 6:**
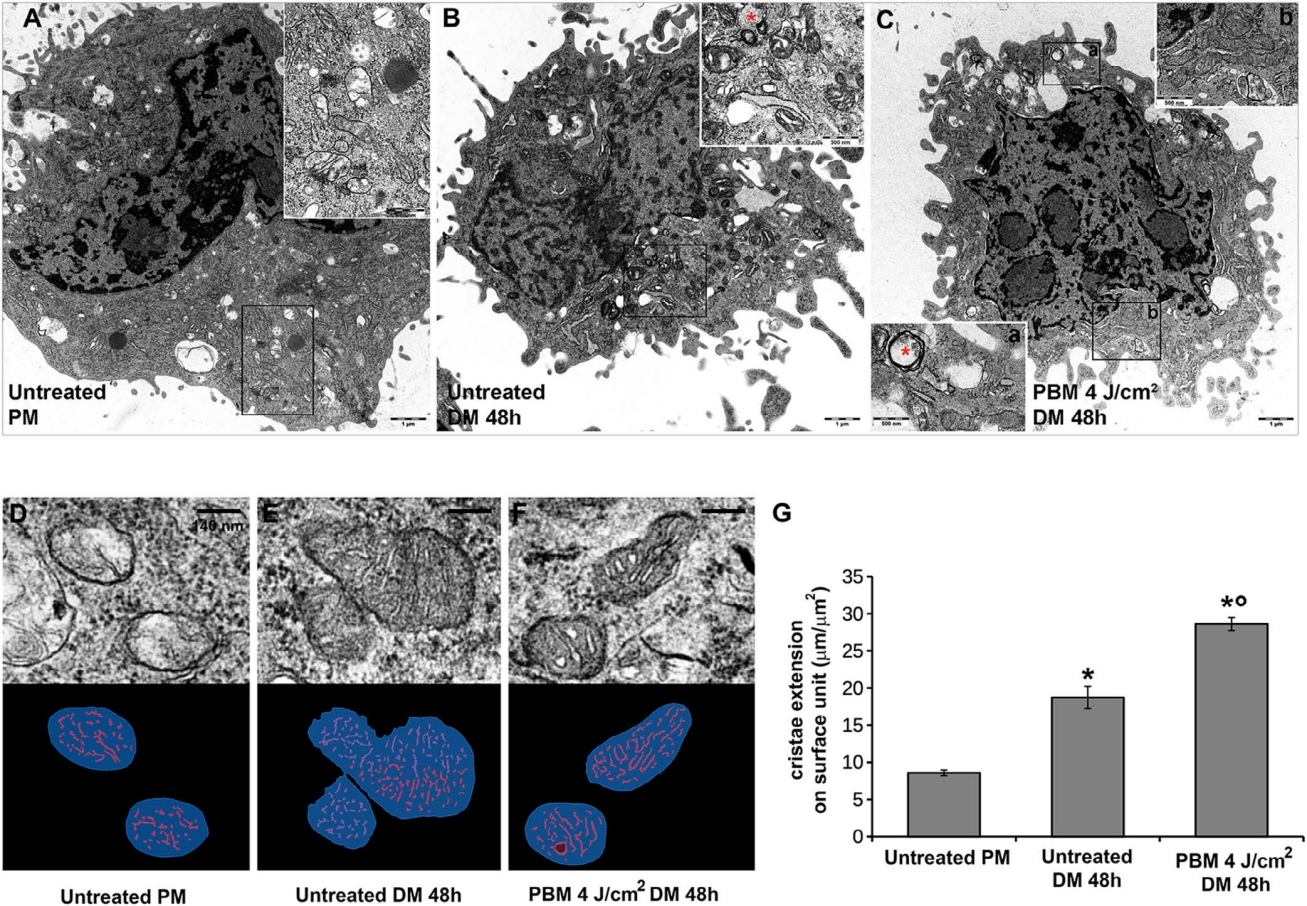
Ultrastructural transmission electron microscopy (TEM) and morphometric analysis of mitochondria. Myoblasts were cultured in proliferation medium (PM) or exposed or not (untreated) to PBM with 4 J/cm^2^ energy density and cultured in differentiation medium (DM) for 48 h. (A–C) Representative images of C2C12 cells in different experimental conditions. Insets in A, B and in C show mitochondria and autophagic vacuoles (red * in B and C) containing myelin‐like structures. (D–F) Identification of mitochondrial sections via TEM. Upper row (left to right): control untreated myoblasts (D), untreated cells cultured in DM for 48 h (E), cells in DM exposed to PBM with 4 J/cm^2^ energy density (F). Lower row: ROIs obtained using the semiautomated protocol for the quantification of mitochondrial section surface area (blue) and mitochondrial cristae overall length (red). (G) Quantitative evaluation of the metabolic activity coefficient (length/area). Data are mean ± SD. *p < 0.001 vs. untreated PM; °*p* < 0.001 vs. untreated DM 48 h (One‐way ANOVA with post hoc Tukey).

Ultrastructural analysis by TEM of the myoblasts under the different experimental conditions (Figure 6) corroborated the results of CLSM and WB analyses. Cells grown in PM appeared to be poorly differentiated (Figure 6A,D). They showed irregularly shaped nuclei and scarce cytoplasm with few organelles, mainly free polyribosomes, scattered rough endoplasmic reticulum (RER) cisternae, small Golgi complexes, several glycogen particles, and some oval‐shaped mitochondria with few cristae. Autophagic vacuoles containing myelin‐like structures were often observed. The myoblasts switched to DM for 48 h showed signs of increased differentiation (Figure 6B,E). Compared with the cells cultured in PM, they were larger and irregular in shape due to several lamellipodes, contained more abundant polyribosomes, RER cisternae, glycogen particles, and rod‐shaped mitochondria with more numerous cristae, a larger Golgi complex, several microfilament bundles, and numerous autophagic vacuoles. Of note, this is not surprising considering that autophagy has been demonstrated to play an essential role during the activation of SCs to generate nutrients that are essential for the generation of ATP and thus to meet the high bioenergetic demands of the activation process [62, 63]. Myoblasts stimulated with red PBM and cultured in DM for 48 h, besides the noted signs of increased differentiation, also showed features of metabolic activation (Figure 6C,F); they were larger and contained abundant polyribosomes, RER cisternae, and multiple nucleoli, indicating increased protein synthesis. Mitochondria were increased in number, had elongated, rod‐like shapes, and contained densely packed cristae, sometimes with a zig‐zag profile typical of hyper‐metabolic striated muscle fibers. Signs of mitochondrial neogenesis by binary scission (separation of the inner chamber in two moieties, mid‐length constrictions) were seldom found.

Machine Learning analysis performed by using AIVIA software aimed to provide morphometric information related to mitochondria cristae surface extension (Figure 6G) relatable to the cell metabolic activity, confirmed the qualitative data from TEM analyses. Indeed, the cristae surface expansion per surface unit increased in the mitochondria of differentiated cells and even more in the mitochondria of the cells subjected to red PBM.

### 3.4 Red PBM (4 J/cm^2^) Stimulates Cell Secretion of EVs With Promyogenic Profile

**FIGURE 7:**
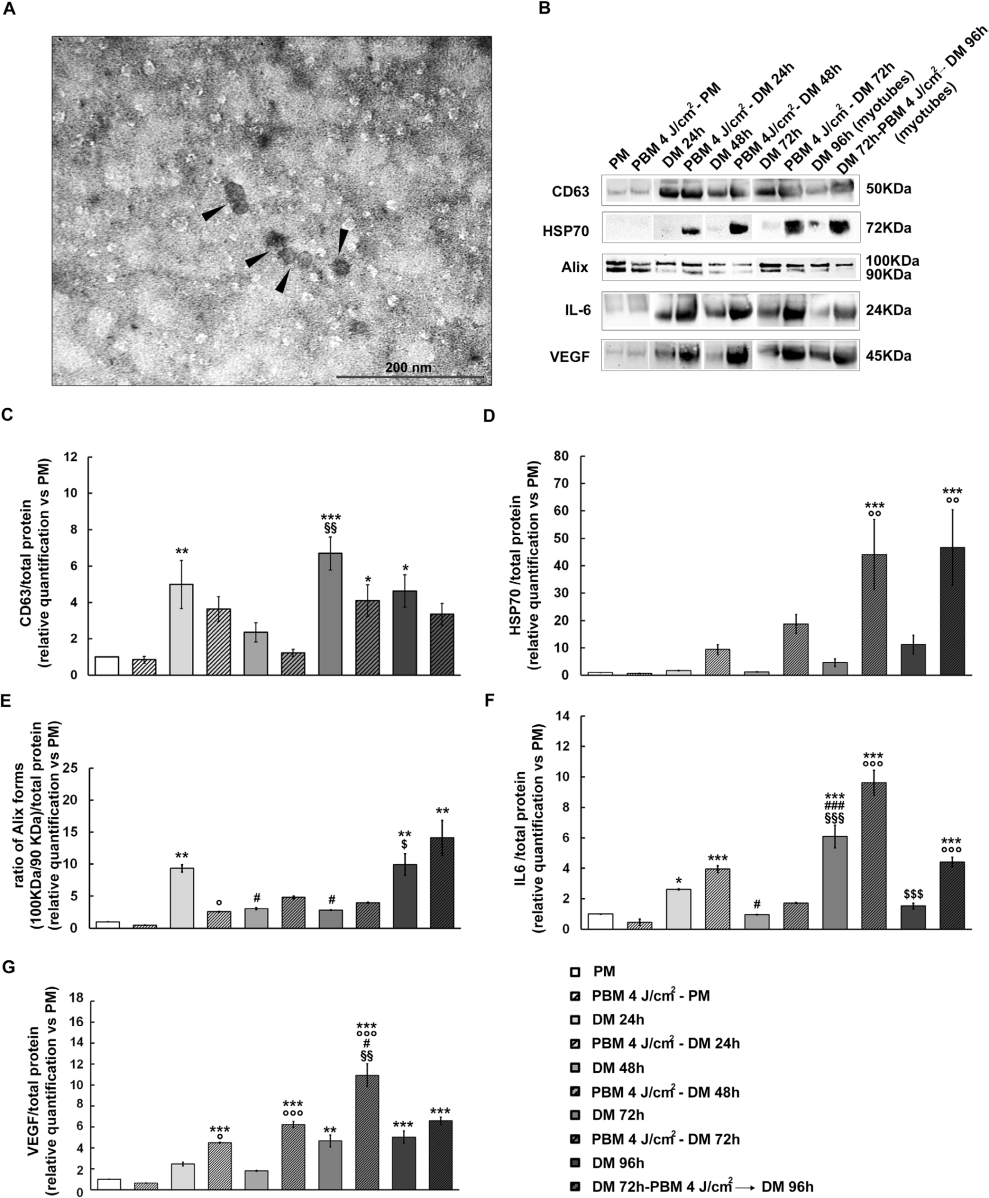
Characterization of extracellular vesicles' (EVs). Morphological and molecular characterization of EVs isolated from conditioned medium by cells exposed or not to PBM with 4 J/cm^2^ energy density and cultured in proliferation medium (PM) or in differentiation medium (DM) for 24, 48, and 72 h. In other experiments EVs were isolated from cells cultured in DM for 96 h or in DM for 72 h in order to form myotubes then exposed to red PBM and cultured for additional 24 h (DM 96 h, myotubes). (A) TEM analysis of EVs (arrowheads). (B) Western Blotting. Representative blot of CD63, Alix, and HSP70 exosome markers and of IL6 and VEGF myogenic markers. (C–G) Quantitative analyses of markers' expression represented as fold change compared to PM. Experimental points are indicated in the legend in G. Data are the mean fold change ± SD obtained from three independent experiments. *p ≤ 0.05, **p ≤ 0.01, ***p ≤ 0.001 vs. PM; °*p* ≤ 0.05, °°*p* ≤ 0.01, °°°*p* ≤ 0.001 vs. not irradiated cells (same culture medium and time). ^#^ *p* ≤ 0.05, ^##^ *p* ≤ 0.01, ^###^ *p* ≤ 0.001 vs. DM 24 h; ^§^ *p* ≤ 0.05, ^§§^ *p* ≤ 0.01, ^§§§^ *p* ≤ 0.001 vs. DM 48 h; ^$^ *p* ≤ 0.05, ^$$^ *p* ≤ 0.01, ^$$$^ *p* ≤ 0.001 vs. DM 72 h (one‐way ANOVA with post hoc Tukey).

Finally, we evaluated how red PBM (4 J/cm^2^) influenced the ability of C2C12 cells to release EVs. Accordingly, we analyzed cells cultured in PM or undergoing myogenic differentiation by culturing in DM (24, 48, and 72 h) or mature myotubes (cells cultured in DM for 72 h, exposed or not to PBM and cultured in DM for further 24 h‐DM 96 h, myotubes). To this aim, total EVs were isolated from cells in different experimental conditions and characterized by TEM (Figure 7A) and WB analysis of the expression of exosome markers CD63, HSP70, and Alix (Figure 7B–G) [51, 53]. TEM analysis demonstrated EVs isolated from cell culture conditioned media with a round shape morphology and a diameter ranging from 30 to 50 nm (Figure 7A). Western blot results showed a strong positive CD63 signal in all samples induced to myogenic differentiation by cell culturing in DM for 24, 48, and 72 h compared to PM (Figure 7B). Of note, differentiated myotubes (DM 96 h) still released a high level of CD63+ EVs (Figure 7B). In particular, quantitative analysis demonstrated an increase of 7‐fold CD63 signal in C2C12 cells cultured in DM for 72 h and a 5‐fold increase of CD63‐positive EVs in fully differentiated myotubes (96 h) (Figure 7C). Cells subjected to red PBM treatment did not show significant differences in CD63 signal compared to untreated ones (Figure 7C).

The expression of HSP70 (Figure 7B,D) was extremely low in proliferating cells (PM). Of note, quantitative analysis demonstrated a 5 fold upregulation of HSP70 in C2C12 cells cultured in DM for 72 h and a 11 fold upregulation in fully differentiated myotubes (DM 96 h) compared to PM samples. Red PBM treatment did not induce significant differences in the HSP70 expression when cells were cultured in PM. By contrast, cells exposed to red PBM and cultured in DM exhibited a robust increase of HSP70 as compared to not irradiated cells (Figure 7D): quantitative analysis demonstrated a 20 fold increase of HSP70 level in C2C12 cells differentiated up to 48 h and a 44 fold increase in C2C12 cells cultured in DM 72 h. Moreover, fully differentiated myotubes exposed to red PBM, showed a 46 fold increase of expression of HSP70.

Alix showed a double band signal corresponding to the two conformations of the protein: the 100 kDa band corresponds to the phosphorylated active open conformation of Alix while the 90 kDa band corresponds to the closed inactive conformation of Alix (Figure 7B) [64]. Quantitative analysis of WB data, represented as the ratio of the signal between the active and inactive forms of Alix compared to PM, showed a 10‐fold increase in the ratio in cells cultured in DM for 24 h and a 15‐fold increase in the ratio in differentiated myotubes (DM 96) (Figure 7E). At DM 48 h and DM 72 h, we observed a decrease in the ratio as compared to DM 24 h. Red PBM did not alter the ratio of Alix band expression except for DM 24 h, where we observed a decrease in the ratio. We next investigated the presence of promyogenic factors such as IL6 and VEGF in the released EVs (Figure 7B,F,G). Results showed an increase in IL6 expression in all the PBM irradiated cells cultured in DM for 24, 48, and 72 h as compared to the respective untreated ones (Figure 7B). In particular, quantitative analysis demonstrated a 10‐fold increase in IL6 in C2C12 myoblasts exposed to red PBM and cultured in DM for 72 h as compared to nonirradiated ones (Figure 7F). A very similar trend was observed in PBM irradiated myotubes (DM 72 → 96 h) compared to untreated ones, where the expression of IL6 in released EVs was 5‐fold higher compared to untreated samples (Figure 7F). As far as the myogenic marker VEGF is concerned, its expression was low in proliferating cells and resulted in increased levels in the cells induced to myogenic differentiation, as well as in mature myotubes (Figure 7B). Quantitative analysis showed a 5‐fold increase in VEGF signal in differentiating cells and fully differentiated myotubes (Figure 7G) compared to proliferating cells. Notably, red PBM treatment strongly induced the upregulation of VEGF in all the cells cultured in DM undergoing myogenic differentiation (upregulation up to 10‐fold in DM treated for 72 h) and in differentiated myotubes (DM 72 h → 96 h) (Figure 7B,G).

## 4 Discussion

PBM is a non‐invasive and painless treatment that may be a promising innovative and alternative approach in the management of skeletal muscle lesions. Indeed, increasing experimental evidence shows the effectiveness of this type of phototherapy to boost skeletal muscle tissue repair essentially by reducing inflammation, limiting the aberrant fibrotic reparative response, while stimulating myofiber regeneration [16, 17, 18, 19, 65, 66, 67, 68, 69, 70].

However, PBM is still viewed skeptically in the field of skeletal muscle regenerative medicine for therapeutic and/or preventive purposes. The main reasons for limiting its use are twofold. The first is represented by the complexity of the technology and the heterogeneity of employed operative parameters. The lack of univocal operating standardized guidelines, in terms of wavelengths, energy and power densities, irradiation mode, as well as time of exposure and other setting parameters, has produced sometimes contradictory outcomes and hampered meaningful comparison of the results [16, 22, 30, 33, 65, 71, 72]. Moreover, it is worth saying that this scenario is complicated by the fact that a biphasic‐dose cellular response to the same wavelength has been frequently observed, with low levels of light that are more effective than the higher ones at stimulating cell functionality [23]. The second reason that may limit its use is due to the cellular and molecular mechanisms underpinning PBM effects, especially those occurring in muscle tissue, that have not been fully established yet [34].

Based on these considerations, this research focused on identifying the effective PBM parameters able to positively impact muscle cell behavior, boosting the differentiation process and improving the healing rate of tissues. This in vitro study aligns with this perspective by investigating the response to red PBM treatments with three different energy densities (0.4, 4, and 8 J/cm^2^) of myoblastic cells induced to undergo myogenic differentiation and of well‐differentiated myotubes, mimicking mature muscle cells.

Our findings have demonstrated the effectiveness of red (635 ± 10 nm) PBM treatment carried out with a diode laser operating in continuous wave and in noncontact mode, in promoting myogenic differentiation of murine myoblasts, particularly at an energy density of 4 J/cm^2^.

The treatment did not alter the viability and morphology of mature cells/myotubes. Our conclusions were supported by morphological, biochemical, and functional experimental evidence. First, contrast phase microscopy analysis revealed that red PBM (0.4 J/cm^2^)–treated cells particularly formed a major number of myotubes that displayed an even increased diameter and fusion index compared to untreated cells. As observed by CLSM, irradiated cells also exhibited more assembled cytoskeletal F‐actin filaments, likely stress fiber‐like structures, i.e., bundles of cross‐linked actin filament and non‐muscle myosin recognized as structure precursors of contractile myofibrils during myofibrillogenesis [73, 74, 75]. Furthermore, PBM enhanced the expression of early (MyoD) and late (myogenin) specific myogenic markers, as judged by CLSM and WB analysis. The promyogenic effect of PBM was substantiated by TEM analysis, unraveling distinctive ultrastructural signs of cell differentiation.

Notably, the PBM ability to boost the acquisition of a more differentiated myogenic phenotype was strongly supported also by the electrophysiological recordings of cell membrane passive properties and ion currents. In particular, the potentiality of PBM to enhance membrane hyperpolarization is noteworthy. In fact, one of the earliest steps in the differentiation of muscular precursors recognized to be critical is the membrane hyperpolarization. This condition is reached thanks to changes in ion channel expression profile, whereas stem cells tend to be more depolarized [76]. As well, the observed increase in inward Ca^2+^ current during differentiation that was further enhanced by PBM is in line with the recently reported observations assessing that PBM therapy in skeletal muscle stimulates Ca^2+^ ion‐sensitive channels, allowing Ca^2+^ ions to enter the cell [16]. This, in turn, causes an increase in intracellular Ca^2+^ concentrations, which can stimulate a cascade of intracellular events implied in several biological functions, such as gene expression. Moreover, the enhancement of Ca^2+^ channel activation can also increase cAMP production; in turn, cAMP‐mediated activation of transcription factors enables protein synthesis and boosts cell differentiation. However, although current stem cell biology largely focuses on gene expression, transmembrane potentials due to transporters and varied ion channel permeability have been demonstrated to be powerful regulators of cellular processes [77, 78], and PBM is indeed able to affect this aspect.

Growing evidence suggests that mitochondria have been identified as the primary site of red light absorption in mammalian cells; hence, they are the preferential targets of PBM mediating its stimulatory effects [34, 35]. In line with these aforementioned reports, here we found that PBM‐treated cells exhibited signs of mitochondrial biogenesis and increased mitochondrial metabolic activity, corroborating mechanistic insight underpinning the promyogenic action of PBM. Indeed, it is well known that during the differentiation of myoblasts to myotubes, mitochondrial biogenesis takes place actively in order to meet the energy demand for myotube elongation and forming multinucleated long myofibers [79, 80].

Our findings are consistent with a number of previous in vitro studies demonstrating the stimulatory and promyogenic effects of red PBM on skeletal muscle cells. Chaudary S. and co‐workers [33] reported that pulsed red LED light (635 nm) stimulation enhanced various functions of C2C12 cells, including proliferation and mitochondrial activity under both normoxic conditions and simulated hypoxia/reoxygenation stress, a condition known to exacerbate wound healing and contribute to chronic injuries. In line with this, Teuschl A. and co‐workers [81] showed that red LED light (630 nm) promoted proliferation of C2C12 cells and accelerated wound closure in an in vitro scratch‐wound model. In another study, Silva LMG. and co‐workers [67] demonstrated that red PBM (685 nm), particularly at an energy density of 4.6 J/cm^2^ (among 2.0, 4.6, and 7.0 J/cm^2^ tested), exerted a protective effect on C2C12 cells against the cytotoxicity induced by snake venom toxins and promoted their differentiation into myotubes; this effect was accompanied by the upregulation of myogenic factors (MyoD and myogenin) and the increase of intracellular ATP content. Ferreira JH. and co‐workers [19] demonstrated that red low‐level laser irradiation (660 nm, 2 J/cm^2^ energy density) induced a transcriptional myotube‐like profile in C2C12 myoblasts. Of interest, Shefer G. and co‐workers [82] revealed that primary myogenic donor cells irradiated with low‐energy laser irradiation with a laser of 632.8 nm (0.6 J/cm^2^) expanded, and when transplanted, survived, and fused with host myogenic cells to form host–donor syncytia, thus enhancing muscle recovery and regeneration.

An interesting and novel finding of this study is related to the effect of red PBM (4 J/cm^2^) on cell secretion of EVs. EVs are a heterogeneous population of biocompatible, nano‐sized secreted vesicles containing many types of bioactive molecules, including proteins, DNAs, RNAs, microRNAs, lipids, and metabolites that play a key role in cell–cell communication and signaling pathways during different processes, including myogenesis [51, 53, 83]. In particular, we found that red PBM promoted the release of EVs with a promyogenic profile by the cells undergoing myogenic differentiation and by differentiated myotubes, as judged by the increase of expression of promyogenic factors, namely, IL6 [55, 56] and VEGF [57, 58].

This result related to EVs' release is consistent with the observed increase of F‐actin with stress fiber assembly in cells subjected to red PBM that is necessary for exocytosis facilitation. In particular, F‐actin has been implicated in the secretion and subsequent uptake of vesicles by increasing the plasma membrane tension [84, 85]. The promyogenic action of red PBM was also supported by the results showing the increased expression of HSP70 both in EVs from irradiated cells induced to myogenic differentiation and from irradiated myotubes. Indeed, HSP70, besides protecting against cellular stress and maintaining proteostasis, has been demonstrated to have a positive influence on myogenic differentiation driving myoblast fusion [86].

As far as Alix (active form) positive EVs concerning our data showed a decrease of the signal in irradiated cells undergoing the early phase of myogenic commitment (24 h) as compared to untreated cells. Given the observed reduction of Alix (active form) during differentiation time (48 h and 72 h), we can speculate that red PBM may induce an acceleration of the myogenic process. Alix has been demonstrated to be involved in the remodeling and repair of injured sarcolemma, resulting in cleavage and shedding of the damaged part of the cell membrane [64, 87]. In this scenario, we may hypothesize that the reduction of Alix during myogenic differentiation may be relatable to cell fusion, and we may also suppose a protective action exerted by red PBM on the cell membrane. Furthermore, since Alix has been demonstrated to be involved in actin cytoskeleton remodeling [88] we may also speculate that the reduction of Alix‐positive EVs from irradiated cells may be correlated with the need of Alix inside the cells for triggering/starting stress fiber‐like assembly during cell differentiation. Further studies are warranted to deeper explain these results and define the role of Alix in skeletal muscle cell differentiation.

How red PBM affected EVs' secretion needs to be clearly demonstrated. Based on the electrophysiological results of this study concerning ion currents, one mechanism regulating the promotion of EV generation/release by PBM may be correlated to calcium increase as observed in other cell types [89, 90, 91]. Future experiments could provide a better understanding.

In conclusion, our study provides experimental evidence supporting the promyogenic effects of red PBM and contributes to defining the most suitable operating PBM dose parameters for potential application in muscle regeneration.

However, translating findings “from bench to bed” to clinical muscle trauma or chronic pathology must be approached with great caution. Indeed, parameters that prove effective in in vitro cell models may not be directly applicable to in vivo tissues/organs. Factors such as tissue specific composition, structural heterogeneity, metabolism, vascular supply, and immune response—absent in simplified in vitro systems—can significantly affect light penetration, dose absorption, bioavailability, and ultimately, therefore, therapeutic outcomes. In vitro, cells are typically cultured in monolayers (single or few layers), allowing light to reach the cells directly with minimal interference, scattering, reflection or absorption. As such, the energy density delivered closely approximates the energy actually absorbed by the cells. In contrast, in vivo, light encounters different tissues, each of them endowed with specific optical features that introduce light scattering, absorption, and reflection fraction. Accordingly, only a fraction of the incident light may effectively reach the target cells [16, 22].

As far as red PBM effects on skeletal muscle in animal models are concerned, it has been shown that red laser irradiation (685 nm, 4 or 4.2 J/cm^2^) was able to significantly decrease venom‐induced myonecrosis in mice [92, 93] and that red laser stimulation (660 nm, 10, 50 J/cm^2^) accelerated the muscle regeneration process in a rat model of muscle cryolesion by increasing MyoD and myogenin gene expression [68].

Again, from a translational perspective, it must always be taken into account that differences in the anatomy and tissue features between small animals and humans affect the dose–response. Moreover, the efficacy of PBM may be significantly affected by the physiological state of injured tissue (e.g., acute versus subchronic/chronic muscle damage context) [16].

However, in vitro models represent a mandatory research phase for offering valuable PBM mechanistic insights determining initially the effective treatment/dosing parameters to be then appropriately applied in in vivo studies and eventually in a clinical setting [14, 21, 94, 95].

Further preclinical and clinical trial studies are warranted to optimize the potential therapeutic value of PBM, determining additional factors that influence the effects and the outcomes of the treatment, to achieve an even higher level of reliability and even to perform a personalized treatment according to patient needs.

## Author Contributions

M. Parigi, A. Tani, G. Teti, M. Falconi, M. Mattioli Belmonte, R. Squecco, F. Chellini, C. Sassoli conceived and designed the research; M. Parigi, A. Tani, F. Palmieri, R. Garella, A. Longhin, G. Teti, D. Nosi, D. Guasti, C. Licini, A. La Contana, R. Squecco, F. Chellini, and C. Sassoli performed the research and acquired the data. All authors analyzed and interpreted the data. All authors were involved in drafting and revising the manuscript.

## Conflicts of Interest

The authors declare no conflicts of interest.

## Data Availability

The data that support the findings of this study are available in the Materials and Methods and Results of this article.
